# Enhanced monitoring of Alzheimer's disease brain atrophy using composite value ratios of volumes

**DOI:** 10.1093/braincomms/fcaf497

**Published:** 2025-12-15

**Authors:** Isaac Llorente-Saguer, Neil P Oxtoby

**Affiliations:** UCL Hawkes Institute and Department of Medical Physics and Biomedical Engineering, University College London, London, WC1E 6BT, UK; UCL Hawkes Institute and Department of Computer Science, University College London, London, WC1E 6BT, UK

**Keywords:** CVR, composite value ratio, ICV, intracranial volume, CU, cognitively unimpaired, CI, cognitively impaired, SSE, sample size estimate

## Abstract

Brain atrophy is a natural consequence of ageing but can be accelerated by neurodegenerative diseases such as Alzheimer’s disease. We apply an existing algorithm to identify new biomarkers that better track volumetric changes over time, i.e. atrophy. These new biomarkers are the volumetric ratio of two composite regions of interest, identified by an algorithm optimized to enhance the monitoring of Alzheimer’s disease progression. The algorithm prioritizes biomarkers with less noisy trajectories (quantified by lower sample size estimates for clinical trials) and that capture disease signal by showing a greater rate of change in amyloid-positive versus amyloid-negative individuals, ensuring the biomarker effectively reflects Alzheimer's pathology. Data from 1381 individuals from the Alzheimer’s Disease Neuroimaging Initiative database having multiple MRI scans were analysed. The new biomarkers outperformed traditional volumetric measures (whole brain, hippocampus and ventricles) across all metrics. This improvement was particularly pronounced in cognitively impaired individuals, where atrophy is more severe. Among the traditional measures, ventricular volume had the best performance. Results suggest that ratios of regional brain volumes could enhance disease progression tracking, as has been shown in other modalities like fluid biomarker ratios and standardized uptake value ratios from positron emission tomography.

## Teaser

Llorente-Saguer and Oxtoby propose novel atrophy biomarkers, reducing clinical trial sample sizes by 75–76% for cognitively impaired individuals and 45–62% for preclinical trials. These biomarkers outperform traditional measures like ventricles, the whole brain or the hippocampus, demonstrating greater efficiency in tracking atrophy independent of amyloid status and cognitive status.

## Graphical Abstract

**Graphical Abstract:**
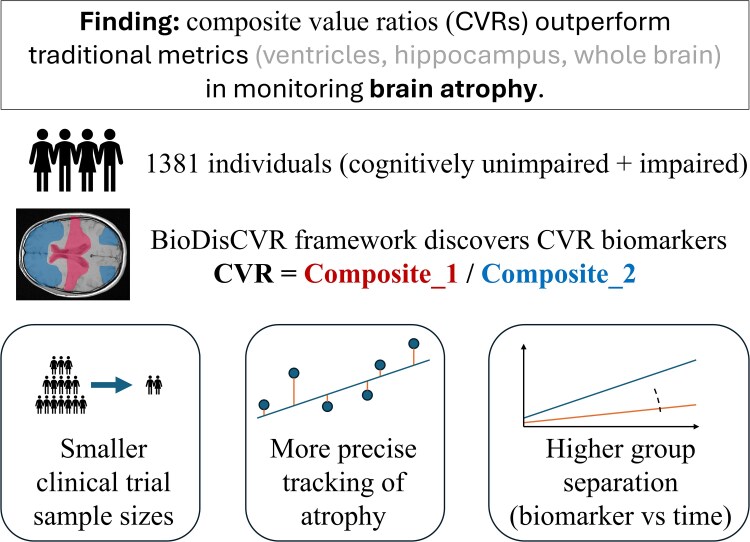

## Introduction

Brain atrophy can result naturally from ageing^1-5^ or be accelerated by neurodegenerative diseases (e.g. Alzheimer’s disease,^3,6-9^ frontotemporal dementia^8^). Understanding volumetric changes can be useful for diagnosis,^6,9^ monitoring disease progression^10^ and evaluating the impact of therapeutic interventions.^11,12^ The following regions are reported to experience a change over time in healthy adults that can be measured with T_1_- or T_2_-weighted magnetic resonance imaging (MRI): whole brain,^1,2,8^ hippocampus,^1,4,9,13^ entorhinal cortex,^13^ ventricles^1-3,5^ and temporal lobe.^1,4^ In Alzheimer’s disease, these are some of the regions of interest that have been reported to have an accelerated and detectable volume change over time with respect to healthy controls: whole brain,^8^ hippocampus,^4^ ventricles,^3^ medial temporal lobe,^4,6^ precuneus^14^ and cingulate cortex.^14^ In Phase 3 randomized clinical trials aimed at intervening on Alzheimer’s disease progression, MRI-based volumetric measures of the whole brain, the ventricles and the hippocampus have been used as primary^11^ and secondary outcome measures.^12,15-17^.

Measurement variance can diminish our ability to detect true changes. Due to the previous success in identifying a data-driven ratio of composite brain regions that improved longitudinal monitoring of tau PET signal,^18,19^ as compared with more traditional biomarkers, we sought to apply the same method to MRI-based regional volumes. It is well known that while some structures experience atrophy, others remain more stable, and the ventricles expand. In fact, specific MRI-based ratio biomarkers, such as the hippocampus-to-ventricle volume ratio (HVR)^20^ or the hippocampal occupancy score (HOC),^21^ have already been proposed to sensitively capture atrophy. Allowing an algorithm to choose the best-performing ratio biomarker among them might improve longitudinal monitoring of changes in the brain due to a neurodegenerative disease, such as Alzheimer’s. Given MRI's wider availability and lower cost compared to tau PET, applying this ratio-based approach to MRI-based regional volumes could vastly enhance the practicality and accessibility of longitudinal monitoring.

## Materials and methods

We apply the BioDisCVR framework^19^ to find a composite value ratio (CVR) aimed at improving the monitoring of brain volume changes due to Alzheimer’s disease. CVR and traditional biomarkers were measured using three main metrics: (i) the sample size needed to detect a 20% change in the gradient in a hypothetical clinical trial; (ii) a measure of longitudinal coherence (translated into a percentage error of the biomarker measure); and (iii) group separation of the biomarker trajectory (according to amyloid positivity (AB) and cognitive status, as two separate analysis). The BioDisCVR framework uses, by default, a genetic algorithm to search for combinations of features (a ratio of aggregate brain regional volumes in this case) that improve a fitness function (described below, in Model). The Genetic Algorithm was configured with elitism set at 2 (the two best CVRs from a generation move on to the next one), a population of 32, a mutation probability of 0.5 and the maximum allowed number of generations was capped at 600, with early stopping if no improvement in the fitness function is achieved in 100 consecutive generations. To increase the robustness of the exploratory model, the genetic algorithm is executed three times with different seeds, and the final CVR is selected as the one that provided the best fitness metric.

### Data

We analysed longitudinal T_1_-weighted (T_1_w) MRI regional volumes from 1381 participants in the ADNI dataset, including all ADNI phases between 2005 and 2025 (ADNI1, ADNIGO, ADNI2, ADNI3, ADNI4), retrieved on 30 May 2025. The segmentation to obtain regional volumes was performed with FreeSurfer 7.1.1.^22^ Both 1.5 and 3.0 T scans were used, without any adjustments for field strength or site (results should be robust to unharmonized data). All T_1_w MRIs passed ADNI's own QC. Table 1 shows the number of subjects and visit counts by cognitive impairment (DX), AB, sex and presence of APOE4 (APOE). The median total follow-up time per individual (earliest visit to latest visit) was 157.6 weeks, with an interquartile range (IQR) of 210.3 weeks (range, 4–809.6 weeks; average, 214.5 weeks; total follow-up time, over 5696 person-years). Participant flow chart reporting exclusion criteria are shown in Supplementary Fig. 1. The ADNI inclusion criteria^23^ were as follows: participants aged between 55 and 90 years (inclusive); having a study partner capable of providing an independent assessment of their functioning; speaking either English or Spanish; willing and able to complete all testing procedures, including neuroimaging; and agreeing to participate in longitudinal follow-up. Additionally, between 20 and 50% of participants had to be willing to undergo two lumbar punctures. The use of certain psychoactive medications was a disqualifying exclusion. Furthermore, the ADNI protocol defined the following cognitive groups, each with specific inclusion and exclusion criteria: cognitively unimpaired (CU) individuals had a Global Clinical Dementia Rating (CDR) score of 0, a memory box score of 0 and a mini-mental state examination (MMSE) score ranging from 24 to 30. They were not depressed, did not have mild cognitive impairment (MCI) as described below and were non-demented. MCI required MMSE scores between 24 and 30, a memory complaint, objective memory deficits (measured by education-adjusted scores on Wechsler Memory Scale Logical Memory II), a CDR of 0.5, no significant impairment in other cognitive areas, preserved daily functioning and the absence of dementia. Alzheimer’s dementia (AD) was considered when the Global CDR was 0.5 or 1.0, the mini-mental state exam score was between 20 and 26 (inclusive), and the NINCDS/ADRDA criteria for probable AD were met. In our study, we consider only the baseline visit diagnosis to emulate a clinical trial scenario. Our CU group are diagnosed in the ADNI dataset as cognitively normal. Our cognitively impaired (CI) group are diagnosed either as MCI or AD.

**Table 1:** Data characteristics

Cognitively impaired, amyloid-positive				
	Total	Subset 1	Subset 2	*P*-value
***N* (subjects)**	564	286	278	
**Age at baseline**	74.0 (7.1)	74.1 (7.1)	73.9 (7.1)	0.801
**Education years**	15.9 (2.7)	15.9 (2.8)	16.0 (2.7)	0.610
**MMSE**	26.6 (2.9)	26.6 (2.8)	26.6 (2.9)	0.823
**Sex (Female)**	247 (43.8%)	125 (43.7%)	122 (43.9%)	0.966
**APOE4 (none)**	178 (31.6%)	91 (31.8%)	87 (31.3%)	0.939

Cognitively unimpaired, amyloid-positive				
	Total	Subset 1	Subset 2	*P*-value
***N* (subjects)**	88	49	39	
**Age at baseline**	73.6 (7.1)	73.9 (6.7)	73.2 (7.6)	0.841
**Education years**	16.5 (2.6)	16.5 (2.6)	16.4 (2.6)	0.959
**MMSE**	29.1 (1.0)	29.2 (1.1)	29.1 (1.0)	0.320
**Sex (female)**	62 (70.5%)	34 (69.4%)	28 (71.8%)	0.811
**APOE4 (none)**	39 (44.3%)	21 (42.9%)	18 (46.2%)	0.837

Amyloid-negative				
	Total	Subset 1	Subset 2	*P*-value
***N* (subjects)**	729	370	359	
**Age at baseline**	71.6 (7.0)	71.7 (7.0)	71.5 (7.0)	0.823
**Education years**	16.6 (2.5)	16.7 (2.5)	16.5 (2.5)	0.291
**MMSE**	28.6 (1.6)	28.6 (1.6)	28.6 (1.6)	1.00
**Sex (female)**	346 (47.5%)	176 (47.6%)	170 (47.4%)	0.954
**APOE4 (none)**	563 (77.2%)	285 (77.0%)	278 (77.4%)	0.932

Demographic and clinical data for the cognitively impaired amyloid-positive (CI, AB+), cognitively unimpaired amyloid-positive (CU, AB+) and amyloid-negative (AB−) cohorts. The data for each group are shown in total and for the subsets derived from a stratified split. Values are presented as mean (standard deviation) or *N* (%). *P*-values, derived from Wilcoxon rank-sum or Chi-squared tests, assess the balance between the training and validation sets for each group, for continuous or categorical variables, respectively.

### Study design

We randomly split the data into two subsets, stratified by the covariates in the model formula below (cognitive status, amyloid status, sex, APOE4 presence, quartiles of age at baseline). The distributions of the two splits are shown in Table 1 and are balanced for all covariate combinations as indicated by the high *P*-values. We report the out-of-sample evaluation metrics of each of the two subsets. In the Statistical analysis section below, we introduce the modelling formula and the evaluation metrics. We consider two cases or target groups: preclinical (CU, amyloid-positive) and clinical (CI, amyloid-positive). We define amyloid-positive individuals as those being amyloid-positive at baseline visit (composite amyloid-PET SUVR above or equal to 0.78 for florbetapir and 0.74 for florbetaben)^24^; due to this missing requirement, 99 (19.1%) CU individuals and 476 (33.1%) CI individuals were excluded.

### Statistical analysis

We fit a linear mixed-effect model with correlated random intercepts and slopes, similar to the framework work on tau PET biomarkers for AD.^19^ Common covariates (age, sex, APOE4, education) are analysed and included in the model where significant (iterative ANOVA tests), using the ventricles divided by the total intracranial volume (ICV) as a baseline biomarker. The ventricles are used because they show the most stable longitudinal change (smaller model residuals), compared to other volumetric biomarkers used in clinical trials (entorhinal cortex, hippocampus or whole brain), as shown in earlier work.^5,25^ Binary variables for AB and cognitive impairment are also included, as their fixed effects will help us evaluate disease signal of biomarkers (positive individuals in either are expected to have more pronounced brain changes than otherwise).

The formula used to fit all groups is the following:


$$\begin{array}{rl} \log\;(biomarker) & =\;\beta_{0}+b_{0}+\beta_{1}\cdot Age.bl+\beta_{2}\cdot sex\\ & \;+\;time(\beta_{3}+b_{1}+\beta_{4}\cdot DX+\beta_{5}\cdot AB\\ & \;+\beta_{6}\cdot APOE4)+\varepsilon \end{array}$$

where the age at baseline visit and sex (female = 1, male = −1) contribute to the intercept, and the gradient (base β3) is modified by cognitive status at baseline visit (DX = 0 for CU, 1 otherwise), AB at baseline visit (AB = 0 when negative, 1 otherwise) and having at least one APOE4 allele (APOE4 = 0 if none, 1 otherwise). We include correlated random effects for the intercept (b_0_) and gradient (b_1_), since this provides invariance to time-shifts,^26^ and we use a relative time point (the average of visit dates, per individual). Education was not included, as it did not have a significant impact on the fitting of the model (*χ*2 = 0.83, DF = 1, *P* = 0.36). The low diversity in ethnicity and race precluded its inclusion in the model: out of the 1381 individuals in the study, over 92% were white (see Supplementary Table 1 for specific numbers). When considering a specific target group for a clinical trial (only amyloid-positive and either CU or CI), we use the same equation, but *AB* and *DX* are removed because these variables become fixed.

We calculate multiple evaluation metrics (exemplified in the Graphical Abstract) to compare biomarkers, namely, sample size estimate (SSE), measurement percentage error (as per our model fitting) and group separation (by AB and cognition), described below.

**Sample size estimate (SSE)** is the SSE per branch (placebo and treatment) for a hypothetical clinical trial designed for 80% power, with 20% treatment effect size, with measurements at the initial and final visit of the trial. Length is 78 weeks (1.5 years) for the clinical trial (CI, amyloid-positive, similar to the trials ENGAGE/EMERGE,^16^ CLARITY-AD,^27^ TRAILBLAZER-ALZ 2^15^) and 234 weeks (4.5 years) for the preclinical group (CU, amyloid-positive, similar to the A4 trial^28^). Given the same trial design conditions, a lower SSE indicates that we can achieve the same detection of effect size with a smaller number of participants. Even if a trial is not based solely on this, we can understand this metric as a sensitivity metric, quantifying how good the biomarker is, accounting for the gradient and variance of the model coefficients and residuals.

As an **error measure**, to quantify the residual variability of the linear mixed-effect model, we define $error=100\text{\%}\;(e^{sd(residuals)}-1)$, where *sd* is the standard deviation and *residuals* are the model residuals. This transformation back to the original scale allows for a more intuitive interpretation of variability in the context of the biomarkers’ actual values. Expressing residual variability as a percentage of the biomarker in its native space facilitates direct comparisons and meaningful insights into model performance, where a lower error means a better fit.

We further provide two **group-separation** metrics, corresponding to the *t*-statistic of the fixed effect of being amyloid-positive (*AB* = 1) and also the *t*-statistic of the fixed effect of being CI (*DX* = 1). This is key to ensuring the biomarker is capturing changes related to the disease. A larger *t*-statistic corresponds to better group separation.

The above metrics are provided with 95% confidence intervals. For error and group separation, the confidence intervals were calculated using the model-based (semi-)parametric bootstrap (*n* = 1000) for mixed models function from the lme4 package,^26^ version 1.1.35.1. Confidence limits correspond to the 2.5th and 97.5th percentiles of the bootstrapped distributions. Confidence intervals for the estimated sample size per group were generated automatically by the *lmmpower* function (longpower v1.0.27), using the method described in Diggle *et al*.^29^

### Model diagnostics and evaluation of multicollinearity

To assess model fit and the validity of underlying assumptions, we conduct several diagnostic checks. Variance inflation factors (VIFs) are computed to evaluate multicollinearity among predictors. Model residuals are visually inspected using residual-versus-fitted plots and quantile–quantile (Q–Q) plots to assess homoscedasticity and normality, respectively.

### Association between biomarkers and cognitive measures

We perform a cross-sectional analysis in our amyloid-positive cohort to evaluate how volumetric biomarkers relate to clinical impairment. From the full dataset, we retain one baseline visit per subject by selecting the earliest scan date. We then define a binary outcome, CDR_bin, indicating unimpaired (global CDR = 0) versus impaired (global CDR >0). Biomarker values are standardized relative to the mean and standard deviation of the CU, amyloid-negative subgroup. We also *z*-score age and education (within the amyloid-positive individuals) and recode sex as a 0/1 indicator for female. For each biomarker, we perform two analyses. First, a logistic regression is fit to model the odds of clinical impairment (CDR_bin ∼ biomarker_z + age_z + female + education_z). We report the odds ratio (OR) per one standard deviation increase in the biomarker, its 95% confidence interval, the model's *P*-value and its discriminative ability via the area under the receiver operating characteristic curve (AUC). Second, a linear regression is fit to the baseline MMSE score (MMSE ∼ biomarker_z + age_z + female + education_z). From this model, we report the standardized regression coefficient (β) and the adjusted *R*^2^ as a measure of variance explained. Analyses are conducted in R (v4.5.0).^30^

Furthermore, we examine the usage of volumetric measures in past Phase 3 clinical trials (the A4 Study,^28^ EMERGE,^16^ ENGAGE,^16^ Clarity AD,^27^ TRAILBLAZER-ALZ 2^15^) and calculate the expected detectable effect size for the same clinical trial configuration (number of participants, trial duration and inclusion criteria), for the different biomarkers.

Finally, as an additional CVR validation and application, we obtained the publicly available A4 trial MRI scans (recently available) and processed them using our established pipeline. We compare the annualized per cent change for each biomarker for subjects with valid follow-up data. This was computed as [(endpoint_value − baseline_value) / baseline_value]/duration_in_years * 100%. To estimate the treatment effect, we fitted an analysis of covariance (ANCOVA) model for each biomarker. The annualized per cent change was the response variable, with the trial group (solanezumab versus placebo) as the primary predictor. All models were adjusted for key baseline covariates: age, ApoE ε4 carrier status (presence versus absence), years of education (dichotomized as ≥13 versus <13 years) and the baseline value of the respective biomarker to increase statistical power and control for regression to the mean. From these models, we extracted the least-squares mean (LS-mean) difference between treatment arms, its 95% confidence interval and the corresponding *P*-value.

### Model

We run the BioDisCVR framework^19^ to obtain a CVR: the ratio of two data-driven composite regions. Each composite region is the average volumetric measurement over a number of selected regions. A genetic algorithm explores combinations of 52 bilateral regions’ volumes defined by the Desikan–Killiany atlas in FreeSurfer 7.1.1 (listed in Supplementary Note 1). Similar to the original framework paper, our algorithm’s fitness function to be maximized includes a group separation metric (for disease signal) and a measure of variability (SSE, described below) and is defined as t_AB/(SSE), where t_AB is the amyloid group separation: *t*-statistic of the fixed effect of being amyloid-positive, fitting the model to all data. This value is capped at 2.6 to prevent convergence towards a Pareto front caused by multiple metrics (t_AB and SSE) and to penalize CVRs that fail to demonstrate sufficient group separation. A threshold of about 2.6 standard deviations corresponds to the upper 0.5% tail of a standard normal distribution, indicating strong statistical separation beyond this point.

SSE represents SSEs per group for a hypothetical clinical trial designed for 80% power, with 20% treatment effect size. Measurements are at the initial and final visit of the trial. Length is 18 months for the clinical trial (CI, amyloid-positive) and 54 months for the preclinical group (CU, amyloid-positive). For the analysis where we fit all groups (regardless of cognitive or amyloid status), we consider a hypothetical clinical trial length of 18 months; this is the experimental configuration used to report both the amyloid and cognitive group separation.

As with the original framework,^19^ a CVR is encoded in a single vector as 0/1/2 respectively meaning numerator/excluded/denominator. The genetic algorithm evolves this vector via the fitness function, retaining regional volumes in a combination that improves the fitness.

### Baselines

Results are compared to volumetric biomarkers that have been used as outcome measures^11,15-17^ in Phase 3 randomized clinical trials aimed at intervening on Alzheimer’s disease progression: whole brain, hippocampus and ventricles. We additionally include the entorhinal cortex, for its strong implication with Alzheimer’s disease.^13^ These volumes of interest are all divided by the concurrent ICV. Lastly, we include a biomarker from the literature, which is the ratio of the hippocampal volume to the ventricles.^20^

### Code availability

The framework code for BioDisCVR, used for biomarker discovery using CVRs, is available in a public GitHub repository under the GNU General Public License v3.0 (https://github.com/isaac-6/biodiscvr).

## Results

We present results in the following structure: evaluation metrics, biomarker regions, model fitting and examination of past clinical trials.

### SSE

Table 2 shows the SSEs for the compared biomarkers, for different groups in each column: all the data (SSE all), only for CI amyloid-positive individuals (SSE CI) and only considering CU amyloid-positive individuals (SSE CU). Parentheses indicate 95% confidence intervals obtained by bootstrapping. We can observe that the CVR biomarkers outperform the conventional regions of interest by a considerable margin in all metrics. In particular, CVR requires a 75–76% smaller sample for a clinical trial (CI), and a 45–62% smaller sample for a preclinical trial (CU), compared with the best volumetric biomarker used in clinical trials (ventricles). We also notice the poor performance of the whole brain, entorhinal cortex and hippocampus. Considering all data, CVR biomarkers also show a much lower SSE (in all CVR cases, 59–67% lower) than the ventricles. The literature biomarker, the hippocampal-to-ventricle ratio, performed notably well, especially in the preclinical group (0–4% with respect to CVR), although it had 12–36% higher SSE than CVR in the clinical group. In practice, if a clinical trial was to be powered by CVR instead of the ventricles, assuming a linear screening and recruitment rate,^31^ the time and number of participants would be cut by 52% and 76%, in a preclinical (CU) or clinical (CI) trial, respectively (using the mean out-of-fold biomarker performances).

**Table 2:** Sample size estimate for a hypothetical clinical trial (80% power, 20% effect size)

Biomarker	SSE all	SSE CI	SSE CU
Subset 1			
CVR_CI	**733** (**580, 954)**	**201 (162, 256)**	84 (58, 135)
CVR_CU	863 (680, 1130)	210 (163, 279)	**45 (34, 63)**
Ventricles	2279 (1761, 3064)	812 (596, 1171)	118 (80, 193)
Whole brain	82.7k (36.9k, 327k)	34.0k (12.0k, 327k)	5483 (1411, 6.8 M)
Hippocampus	42.4k (20.6k, 134k)	9110 (4740, 24.2k)	5670 (1097, 75.8k)
Entorhinal	23.6k (12.7k, 57.6k)	4209 (2460, 8790)	1535 (577, 11.3k)
Ventricles/hippocampus	797 (628, 1045)	225 (176, 299)	47 (36, 65)
Subset 2			
CVR_CI	**578 (454, 761)**	**291 (214, 417)**	**80 (53, 135)**
CVR_CU	1084 (793, 1570)	600 (386, 1055)	82 (54, 140)
Ventricles	1435 (1088, 1977)	1238 (790, 2215)	149 (87, 318)
Whole brain	117k (46.0k, 727k)	60.8k (21.9k, 544k)	1594 (435, 218k)
Hippocampus	36.7k (17.1k, 128k)	9129 (4723, 24.6k)	1079 (327, 32.2k)
Entorhinal	17.1k (9408, 40.7k)	4182 (2360, 9353)	921 (309, 12.3k)
Ventricles/hippocampus	730 (562, 985)	395 (277, 609)	81 (53, 138)

The sample size estimate (SSE) is found by fitting a linear mixed effects model to the target group: all (for reference), cognitively impaired (CI) or cognitively unimpaired (CU). Our biomarkers (CVR) were identified using the other split in which they are evaluated. CVR_CI was found by minimizing the sample size estimate for the cognitively impaired (CI), while CVR_CU was focused on the cognitively unimpaired group (CU). In bold, the best result per subset. Hippocampus, ventricles, entorhinal cortex (EC) and whole brain are each divided by intracranial volume (ICV). The last biomarker of each group is the ventricles divided by the hippocampus (bilateral).

### Percentage error

Next, we examine the percentage error in Table 3. As in Table 2, Table 3 shows results for different groups: all the data (all), only for CI amyloid-positive individuals (CI) and only considering CU amyloid-positive individuals (CU). Parentheses indicate 95% confidence intervals obtained by bootstrapping. These results show that the CVR biomarkers vastly outperform the conventional regions of interest in all the analyses. In particular, in the evaluation with unseen data, CVR has a 67–77% smaller error in a clinical trial setting (CI), and a 1–60% smaller error for a preclinical trial (CU), compared with the ventricles (very modest in Subset 2). We also notice the large variance of the entorhinal cortex. When evaluating all data, CVR biomarkers also outperform traditional regions of interest (37–75% smaller error). Compared with the hippocampal-to-ventricle ratio, CVR has a 19–53% smaller percentage error.

**Table 3:** Percentage error

Biomarker	Error all	Error CI	Error CU
Subset 1			
CVR_CI	**1.75 (1.70, 1.81)**	**1.77 (1.69, 1.86)**	**1.64 (1.43, 1.90)**
CVR_CU	3.68 (3.58, 3.80)	2.62 (2.50, 2.75)	2.36 (2.06, 2.73)
Ventricles	7.31 (7.10, 7.53)	7.86 (7.47, 8.27)	6.04 (5.25, 6.93)
Whole brain	6.64 (6.43, 6.85)	8.13 (7.77, 8.57)	5.77 (4.96, 6.45)
Hippocampus	6.89 (6.69, 7.12)	8.44 (8.03, 8.89)	6.11 (5.25, 6.88)
Entorhinal	9.45 (9.16, 9.75)	10.89 (10.37, 11.45)	8.34 (7.26, 9.39)
Ventricles/hippocampus	4.39 (4.27, 4.52)	3.85 (3.67, 4.05)	3.49 (3.02, 4.03)
Subset 2			
CVR_CI	**2.16 (2.09, 2.23)**	**2.29 (2.18, 2.41)**	1.94 (1.64, 2.27)
CVR_CU	3.45 (3.34, 3.56)	3.62 (3.44, 3.79)	2.81 (2.34, 3.31)
Ventricles	5.51 (5.34, 5.67)	6.96 (6.62, 7.31)	2.85 (2.43, 3.34)
Whole brain	5.07 (4.93, 5.23)	6.56 (6.22, 6.83)	**1.15 (0.97, 1.35)**
Hippocampus	5.63 (5.46, 5.81)	7.31 (6.93, 7.68)	2.18 (1.83, 2.57)
Entorhinal	8.42 (8.16, 8.69)	9.84 (9.36, 10.33)	6.35 (5.34, 7.48)
Ventricles/hippocampus	3.87 (3.74, 3.99)	4.19 (3.95, 4.38)	3.50 (2.94, 4.14)

Evaluation of the percentage error for the different biomarkers, in both splits of the data (Subsets 1 and 2). Our biomarkers (CVR) were identified using the other split in which they are evaluated. CVR_CI was found by minimizing the sample size estimate for the cognitively impaired (CI), while CVR_CU was focused on the cognitively unimpaired group (CU). In bold, the best result per subset. Hippocampus, ventricles, entorhinal cortex (EC) and whole brain are each divided by intracranial volume (ICV). The last biomarker of each group is the ventricles divided by the hippocampus (bilateral).

### Group separation

Table 4 shows the group separation for AB and cognitive status. As described in Materials and Methods, we report the *t*-statistic of the fixed effect of being amyloid-positive and also the *t*-statistic of the fixed effect of being CI. Parentheses indicate 95% confidence intervals obtained by bootstrapping. Here as well, we can observe that the CVR biomarkers outperform the conventional regions of interest. Ventricles have the next best performance, followed by the entorhinal cortex and hippocampus, with the whole brain having the worst results. In particular, in the evaluation with unseen data, CVR has a 20–101% higher separation regarding AB and a 37–103% higher separation regarding cognitive impairment, compared with the ventricles. The hippocampal-to-ventricle ratio has comparable separation metrics with respect to CVR.

**Table 4:** Group separation, both in amyloid positivity and cognitively

Biomarker	Amyloid positivity separation	Cognitive group separation
Subset 1		
CVR_CI	**9.84 (7.91, 11.93)**	**4.11 (2.21, 6.08)**
CVR_CU	9.39 (7.37, 11.44)	3.87 (1.96, 5.97)
Ventricles	7.79 (5.86, 9.90)	2.02 (0.15, 3.94)
Whole brain	1.99 (0.01, 3.92)	0.72 (−1.21, 2.82)
Hippocampus	3.17 (1.21, 5.02)	1.61 (−0.29, 3.57)
Entorhinal	4.74 (2.97, 6.92)	1.18 (−0.80, 3.00)
ventr_hippocampus	9.53 (7.45, 11.50)	3.87 (1.91, 5.86)
Subset 2		
CVR_CI	**8.49 (6.46, 10.44)**	2.08 (0.15, 4.10)
CVR_CU	6.75 (4.68, 8.86)	2.25 (0.34, 4.26)
Ventricles	4.22 (2.45, 6.20)	1.51 (−0.51, 3.47)
Whole brain	3.42 (1.47, 5.32)	0.34 (−1.70, 2.33)
Hippocampus	5.53 (3.43, 7.63)	0.25 (−1.68, 2.32)
Entorhinal	4.42 (2.42, 6.60)	1.02 (−0.90, 3.15)
ventr_hippocampus	7.83 (5.83, 9.91)	**2.48 (0.55, 4.46)**

The separation is given by the *t*-statistic of the fixed effect of being amyloid-positive or cognitively impaired, fitting a linear mixed-effect model to a split of the data (Subsets 1 and 2). Our biomarkers (CVR) were identified using the other split in which they are evaluated. CVR_CI was found by minimizing the sample size estimate for the cognitively impaired (CI), while CVR_CU was focused on the cognitively unimpaired group (CU). The negative separation indicates the direction of the difference between groups. In bold, the best result per subset. Hippocampus, ventricles, entorhinal cortex (EC) and whole brain are each divided by intracranial volume (ICV). The last biomarker of each group is the ventricles divided by the hippocampus (bilateral).

The detailed region list of the CVR biomarkers aimed at reducing the SSE for a clinical trial involving CI, amyloid-positive individuals is shown in Table 5 (Cognitively impaired). In the numerator, both biomarkers found in the two splits of the data: the ventricles, frontal pole, isthmus of cingulate gyrus, the caudal anterior cingulate cortex and the mid-posterior and central part of the corpus callosum. In the denominator, they shared the hippocampus, amygdala, entorhinal cortex, thalamus, nucleus accumbens, ventral diencephalon and the anterior part of the corpus callosum.

**Table 5:** Regions of the biomarkers discovered in each subset

Cognitively impaired			
Numerator		Denominator	
Subset 1	Subset 2	Subset 1	Subset 2
**cc_mid_posterior**	**cc_mid_posterior**	**cc_anterior**	**cc_anterior**
**cc_central**	**cc_central**	**entorhinal**	**entorhinal**
**caudalanteriorcingulate**	**caudalanteriorcingulate**	**thalamus**	**thalamus**
**isthmuscingulate**	**isthmuscingulate**	**hippocampus**	**hippocampus**
**frontalpole**	**frontalpole**	**amygdala**	**amygdala**
**lateral_ventricle**	**lateral_ventricle**	**accumbens_area**	**accumbens_area**
parsopercularis	brainstem	**ventraldc**	**ventraldc**
	lingual	cc_posterior	transversetemporal
	posteriorcingulate	bankssts	choroid_plexus
		parahippocampal	
		pallidum	

Cognitively unimpaired			
Numerator		Denominator	
Subset 1	Subset 2	Subset 1	Subset 2
**lateral_ventricle**	**lateral_ventricle**	cc_mid_anterior	bankssts
temporalpole	cc_posterior	isthmuscingulate	parstriangularis
	cc_central	lateralorbitofrontal	pericalcarine
	cc_anterior	paracentral	temporalpole
		parsorbitalis	cerebellum_cortex
		rostralmiddlefrontal	thalamus
			amygdala
			accumbens_area
			ventraldc

Subset 1/2 indicates the subset of data that was used for the discovery of the biomarker. In black are regions that were selected in both subsets of the data.

The detailed region list of the CVR biomarkers aimed at reducing the SSE for a preclinical trial involving CU, amyloid-positive individuals is shown in Table 5 (Cognitively unimpaired). Both biomarkers found in the two splits of the data included the ventricles in the numerator. In the denominator, they shared no regions.

In both clinical trial scenarios above, the numerator included the ventricles. The most notable differentiator between the trial scenarios is the greater regional consistency with the CI group.

**Figure 1:**
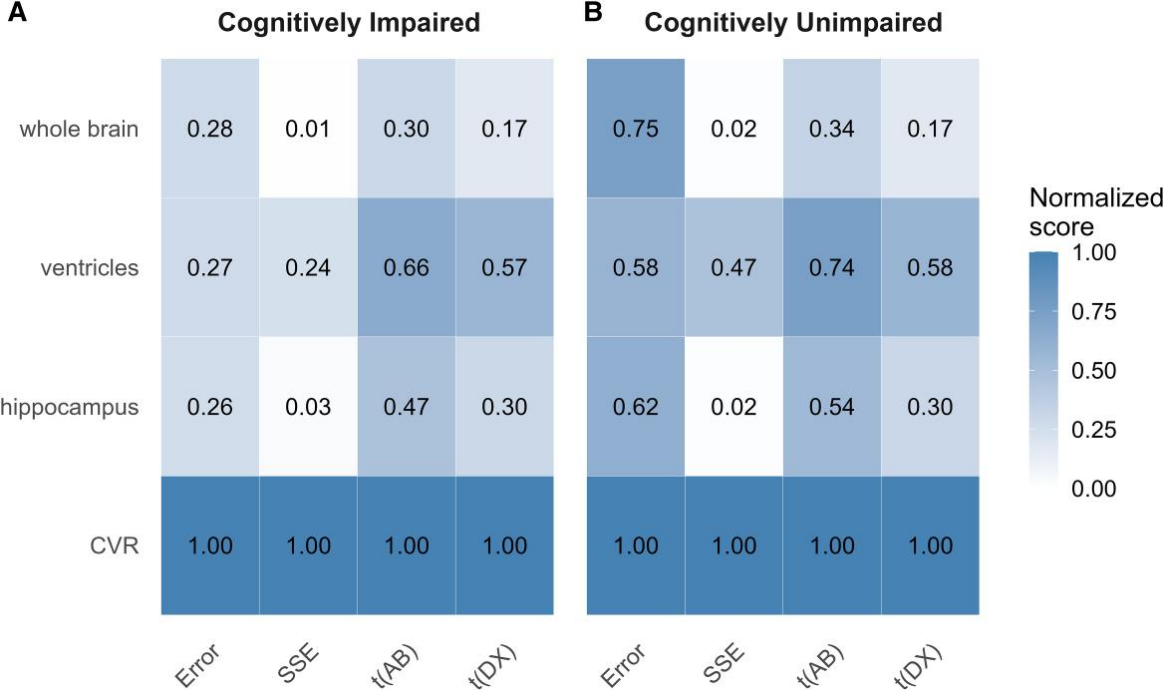
**Summary metrics.** Heatmap of normalized performance scores (0 = worst, 1 = best) for four MRI biomarkers—CVR and the traditional measures hippocampus, whole brain and ventricles—across four evaluation metrics. SSE (sample size estimate for 80% power at 20% effect size), Error (percentage error, defined as SD of model residuals on log-transformed data), t(AB) (*t*-statistic for the longitudinal slope in amyloid-positive individuals) and t(DX) (*t*-statistic for the longitudinal slope in CI individuals). Panels show (**A**) CI (*N* = 564) and (**B**) CU (*N* = 88) amyloid-positive participants. Colour intensity (white to blue) reflects the normalized score, and each cell is annotated with its mean value across the two out-of-fold evaluations.

In Fig. 1, which represents a heatmap of average out-of-fold evaluations (normalized to the best-performing biomarker, per metric), CVR outperformed all traditional volumetric biomarkers across every metric in both diagnostic groups. Among the traditional measures, SSE were best for the ventricles, in both CU and CI groups; group separation (t_AB and t_DX) was best for the ventricles as well, with the hippocampus following, and the whole brain having the worst separation metrics (closer to zero); regarding the error metric, the traditional biomarkers had similar performances in the CI group, but the whole brain performed best in the CU group. These results highlight CVR’s uniformly superior trial efficiency, precision and longitudinal sensitivity compared to standard structural biomarkers used in clinical trials.

**Figure 2:**
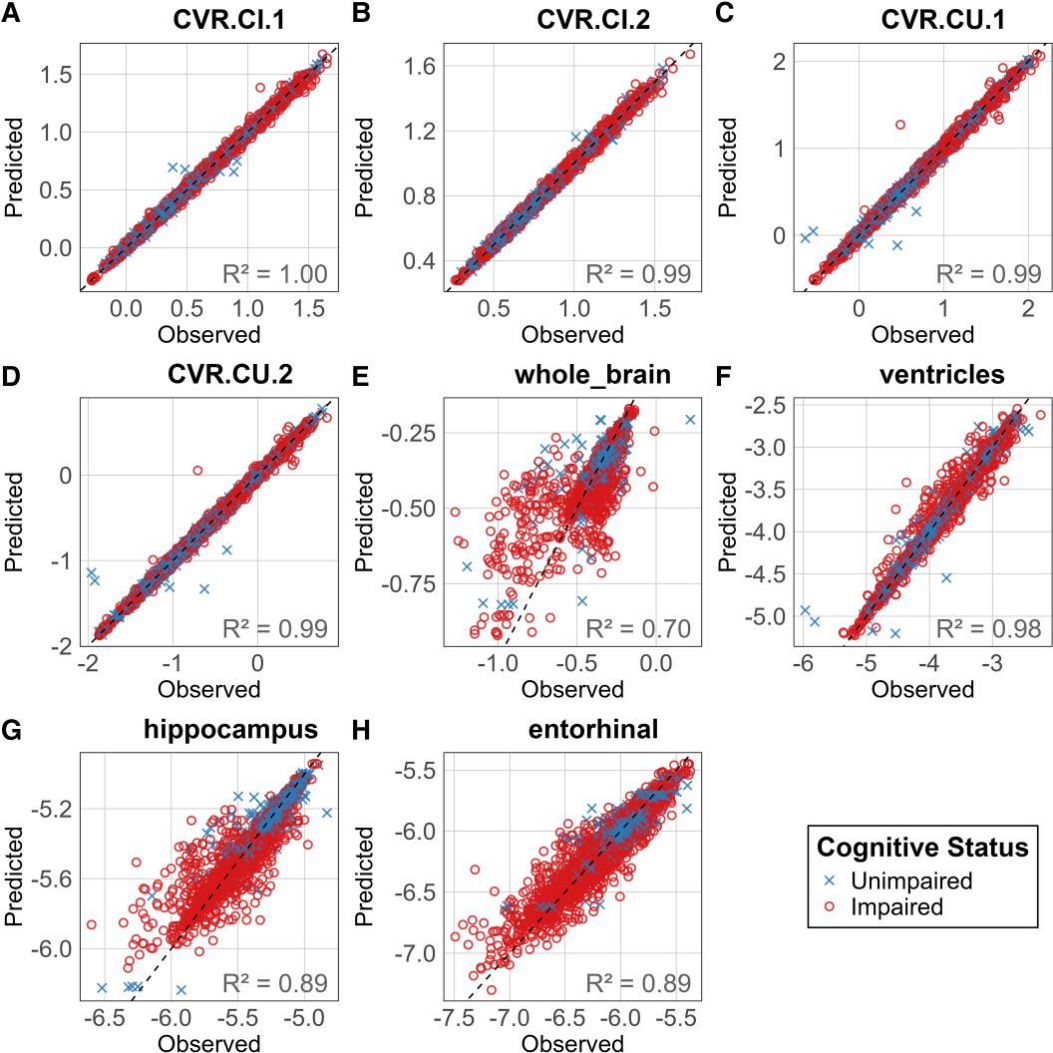
**Predicted versus observed biomarker values (log-transformed), using all data across both discovery subsets.** Each point represents a single scan (*N* = 564 CI; *N* = 88 CU, amyloid-positive). CVR denotes the composite value ratio of two data-driven regions of interest; all other biomarkers are normalized by ICV. CVR naming follows: CI = conditioned to minimize the sample size estimate of CI individuals; CU = conditioned to minimize the sample size estimate of CU individuals; Suffix 1 or 2 indicates the discovery subset. The coefficient of determination (*R*², squared Pearson correlation) is reported in the bottom-right corner of each subpanel.

Figure 2 visualizes biomarker variance as model-predicted versus observed log(biomarker) values, using all data (both subsets). CVR shows a visually narrower spread compared to other biomarkers (in line with the error reported in Table 3), suggesting that it explains longitudinal data better. Of the traditional volumetric biomarkers, ventricles again outperform hippocampus and whole brain. We observe the same results in out-of-sample experiments on the two data subsets, shown in Supplementary Figs. 2 and 3, for data Subsets 1 and 2, respectively.

We next report results of our post hoc checks. For all the biomarkers, none of the VIFs were above 2, suggesting we do not have a strong collinearity problem in our model.^32^ For all biomarkers, the spread of the residuals with respect to the fitted values is a single cluster aligned at zero, showing homogeneity of variance. In Q–Q plots, we observe that the central part of the distribution aligns with the expected quantiles, but the tails do not, which suggests the presence of extreme values. To assess the robustness of our primary mixed-effect model, we conducted a sensitivity analysis on influential data points. Following the visual identification of deviations in Q–Q plots of residuals, we formally identified individuals associated having extreme Pearson residuals (absolute value >3). These individuals were excluded (11 amyloid-positive CU and 41 amyloid-positive CI), and the model was refit on the remaining data. The model's findings were highly robust to the exclusion of these individuals. All fixed-effect estimates shifted by less than one standard error of the original model (range of change: 0.08 to 0.78 standard errors). Furthermore, the 95% confidence intervals for all key predictors, including time and the time:APOE interaction, showed substantial overlap between the original and trimmed models, confirming that our primary inferences are not driven by these extreme observations.

Supplementary Table 2 reports the detectable effect size using past clinical trials’ inclusion criteria and configuration. We observe a considerable difference between biomarkers, and their performance from best to worst is CVR, ventricles, entorhinal cortex, hippocampus and whole brain. To aid readability, we summarize results for the trials targeting CI individuals as CI (EMERGE,^16^ ENGAGE,^16^ Clarity AD,^27^ TRAILBLAZER-ALZ 2^15^) and the one trial targeting CU individuals as CU (A4 Study^28^). Here we summarize the evaluation of the data subsets, to ensure that our reported biomarkers are blindly evaluated, but the table also shows the evaluation using all data. CVR always performed the best, with a detectable effect size below 12% in CI, and around 7% for CU. Ventricles performed second best, with effect sizes between 16–34% in CI and 8% in CU. The entorhinal cortex could detect effect sizes of 22–63% for CI and 24–30% for CU. The hippocampus could detect effect sizes of 30–124% for CI and 26–34% for CU. Last, and worst, the whole brain could detect effect sizes of 60–291% in CI and 29% in CU.

Association between biomarkers and cognitive measures is shown in Supplementary Table 3. Across all biomarkers (baseline and proposed), higher atrophy was significantly associated with increased odds of clinical impairment. All biomarkers demonstrated highly significant relationships with both CDR status (all *P* < 5 × 10⁻³) and continuous MMSE score (all *P* < 5 × 10⁻⁶).

Running the algorithm repeatedly on random subsets of the data (50% of the data, 100 times), we can analyse some regional tendencies of being picked as numerator, denominator or skipped. Supplementary Figs 4 and 5, for CI and CU, respectively, show the stacked bar chart per brain region of the CVR placement.

Regarding the A4 trial MRI data,^28^ we compared the annualized per cent change between the solanezumab (*N* = 401) and placebo (*N* = 423) arms for each biomarker using ANCOVA models (Supplementary Table 4). Our CVRs consistently demonstrated larger, more directionally favourable treatment effects than standard volumetric measures, suggesting superior sensitivity to atrophy in a clinical trial and validating our algorithm’s utility. Specifically, the CVR.CU.1 biomarker (discovered in ADNI’s Subset 1) showed the largest numerical slowing of atrophy, with the solanezumab group exhibiting a 0.28% smaller annualized decline than the placebo group (LS-mean difference = −0.28%, 95% CI [−0.67, 0.12], *P* = 0.17). The CVR.CU.2 measure (discovered in ADNI’s Subset 2) showed a similar trend (difference = −0.20%, 95% CI [−0.59, 0.19], *P* = 0.32). In contrast, the effect on traditional volumetrics was negligible; for example, the adjusted difference for hippocampus/ICV was nearly zero (0.01%, 95% CI [−0.17, 0.18], *P* = 0.95).

We systematically tested for the influence of acquisition site by modelling site as a random effect, separately for each diagnostic group. We conducted likelihood ratio tests for both a main effect of site (baseline differences) and a site-by-time interaction (differences in rate of change) for each of our CVR biomarkers. After applying a Bonferroni correction to account for multiple comparisons within each of these four families of tests, no biomarker showed a statistically significant effect of site (all *P*_adjusted >0.20). This comprehensive analysis provides evidence that site-level variance is not a significant confounder for our primary findings.

The convergence for the different CVRs took 23 to 128 generations of the genetic algorithm, at approximately 3.2 s per generation, using 16 cores of a 12th Gen Intel® Core™ i7-12700H.

## Discussion

This study introduces and validates an approach to monitoring brain atrophy in the context of Alzheimer's disease by employing data-driven CVRs of regional brain volumes derived from MRI, thanks to the BioDisCVR framework. Our findings robustly demonstrate that these data-driven CVR biomarkers significantly outperform traditional volumetric measures—including whole brain, hippocampus, entorhinal cortex and ventricular volume—across multiple key metrics relevant to clinical trial design and disease monitoring. Specifically, CVRs consistently exhibited lower SSEs, reduced measurement error (as per the linear mixed-effect model fitting) and enhanced group separation between amyloid-positive and amyloid-negative individuals, as well as between CU and impaired individuals. The CI group, who are at more advanced stages of the disease, exhibited the most pronounced performance advantage.

Considering SSE, the advantage of CVR biomarkers is particularly notable. Our analysis revealed that CVRs require a substantially smaller sample size to achieve the same statistical power in hypothetical clinical trials compared to traditional volumetric measures. This translates to potentially considerable cost- and time-savings in clinical trials where brain volume is a primary outcome, with the additional benefit of fewer people being exposed to placebo or experimental treatments. Conversely, if the biomarker is not a limiting factor, improved precision augments sensitivity to smaller effect sizes, thereby permitting more nuanced evaluations of therapeutic impact. Furthermore, beyond the area of clinical trials, a biomarker with greater precision can offer benefits in multiple areas, such as disease progression tracking, patient stratification for subtyping, or earlier diagnosis and prognosis. SSEs decrease with longer trial durations, following an approximately inverse relationship. As follow-up length increases, the accumulating divergence in group trajectories relative to within-subject variability enhances the ability to detect treatment effects. Accordingly, the SSE for the 4.5 year CU trial is substantially lower than for the shorter 1.5 year CI trial (Table 2). This reflects not only the statistical advantage of extended follow-up but also the fact that minimal brain atrophy is expected over short intervals—approximately 0.5% per year^33^—a magnitude comparable to the scan-rescan variability of brain volumetry.^34^

In terms of longitudinal measurement of **Error**, CVR biomarkers again demonstrated clear superiority. The percentage error, representing the residual variability of the linear mixed-effect models, was consistently and considerably lower for CVRs compared to traditional measures across all analyses, expect for the whole brain in the Subset 2. A reduced measurement error signifies a more precise and reliable assessment of longitudinal volume changes, crucial for accurate disease monitoring and tracking treatment effects. However, minimizing error alone is not sufficient, as we also need a good disease signal over time.

Regarding **Group Separation**, CVRs consistently provided enhanced differentiation between groups relevant to AD pathology. This improved group separation indicates that CVRs are more effective at capturing the disease signal, distinguishing individuals based on key AD-related characteristics. This enhanced ability to discriminate between groups is vital for biomarker validation, for stratifying patients in clinical trials and potentially for use in diagnostic applications in memory clinics.

The regions selected for CVR differed substantially between CI and CU individuals, both in anatomical distribution and consistency across data subsets. In the CI group, biomarker components were more reproducible and anatomically cohesive, consistently involving limbic and subcortical structures such as the hippocampus, thalamus and amygdala. These regions are closely associated with known patterns of neurodegeneration in Alzheimer’s disease and likely reflect converging structural changes that support stable biomarker identification in symptomatic individuals. In contrast, the CU group yielded more variable biomarker compositions, with less overlap between discovery subsets and a broader distribution across cortical association areas, including the temporal pole, lateral orbitofrontal cortex and cerebellar cortex. This diffuse and heterogeneous pattern in the CU group may reflect greater inter-individual variability in early or preclinical stages, where structural alterations are subtler and less spatially constrained.

### Consistently identified CVR regions in the CI group

Across 100 iterations of BioDisCVR applied to randomly generated subsamples (50% of the cohort each), we observe a robust pattern of region selection within the CI group (see stacked bar chart in Supplementary Fig. 4). As a denominator, the hippocampus was chosen 100% of the time, followed by the entorhinal cortex (97%), nucleus accumbens (94%), amygdala (93%), thalamus (87%), ventral diencephalon (85%) and anterior corpus callosum (82%). This hints at their usefulness in terms of volumetric change, most of which are well known in the literature to be related to AD (entorhinal cortex,^13^ hippocampus,^4,13,35-38^ nucleus accumbens,^38^ thalamus,^39^ anterior corpus callosum,^40^ ventral diencephalon, amygdala^35,36^). In terms of numerator selection, the lateral ventricles are the most consistently chosen (100% of the time), underscoring their sensitivity to pathological expansion. Also frequently selected are the frontal poles (89%), isthmus of the cingulate gyri (81%) and mid-posterior corpus callosum (75%), which could add stability to the overall CVR measure. Regions with greater variability but still chosen over 50% of the time were the caudal anterior cingulate gyrus (62%), posterior cingulate cortex (60%) and central corpus callosum (59%).

### Consistently identified CVR regions in the CU group

Across 100 iterations of BioDisCVR applied to randomly generated subsamples (50% of the cohort each), we observe the ventricles being chosen every single time as numerator (Supplementary Fig. 5) and the central corpus callosum 54% of the time, followed by the choroid plexus (47%). In the denominator, we observe the nucleus accumbens being chosen 82% of the time, and next there’s the banks of the superior temporal sulci being chosen 55%, followed by the rostral middle frontal gyrus (49%), thalamus (46%) and anterior corpus callosum (45%).

Other volume-based biomarkers from the literature have been proposed, such as the hippocampal-to-ventricle ratio.^20^ Our data-driven approach confirms their utility, especially in the CI group, but it also includes other regions that further enhance the biomarker's power, as shown by the lower SSEs. Future research could explore beyond simple volumetric ratio biomarkers (e.g. BSI,^41^ normative modelling,^42^ self-normalizing composite ratios^43^) and consider multimodal approaches, such as including PET. In this case, there will likely be a trade-off between performance and practicality, as PET scans are less readily available and involve higher costs.

The detectable effect size differences are considerable, using configurations of past clinical trials. It should be noted that our evaluated biomarkers were designed for a wider population, whereas the clinical trials’ inclusion criteria had various restrictions (MMSE, CDR, age).

As an additional check, we conducted a sensitivity analysis excluding participants with extreme Pearson residuals (resid >3): 11 amyloid-positive CU and 41 amyloid-positive CI cases. Re-fitting the model on the reduced sample led to shifts in fixed-effect estimates of less than one original standard error, and the 95% confidence intervals for both the time effect and the time:APOE4 interaction continued to overlap closely with those from the full data. These results suggest that our longitudinal findings and the moderating role of APOE4 are not driven by a few atypical trajectories, supporting their applicability to the wider cohort.

Although our primary aim was to derive biomarkers with maximal sensitivity to longitudinal change, we performed a secondary cross-sectional analysis to confirm that these volumetric signatures also map onto baseline clinical status. While our biomarkers were identified within a single cognitive group—either unimpaired or impaired—we assess their associations across the full spectrum of cognition. In our cohort of 652 amyloid-positive participants, all tested biomarkers demonstrated strong and highly significant associations with both dichotomous clinical impairment (global CDR >0) and MMSE scores. Classic biomarkers of focal atrophy, specifically hippocampal and entorhinal volumes, emerged as the most powerful individual predictors (AUC = 0.73–0.76; R² ≈ 0.17–0.19). Notably, our top-performing data-driven biomarker, CVR.CI.2 (AUC = 0.719; R² = 0.168), performed on par with these gold-standard regions of interest, validating that our automated discovery framework can identify clinically relevant composite measures. In contrast, global atrophy measures such as whole-brain volume and ventricular expansion yielded more modest, albeit significant, discrimination (AUC ≈ 0.67; R² ≤ 0.11), underscoring the superior value of targeted atrophy signatures. While these results robustly establish the concurrent validity of the biomarkers, this cross-sectional analysis does not address their prognostic utility. A critical next step, therefore, is to evaluate these markers against longitudinal clinical milestones, such as the conversion from MCI to dementia, to fully characterize their predictive power. In that case, the discovery framework could be updated to include these metrics in the fitness function.

We hypothesize that the superior performance of CVRs might be due to their inherent capacity to normalize for inter- and intra-subject variability and global factors, thanks to their ratio-based construction. This normalization may reduce noise and amplify the signal specific to regional volume changes associated with AD pathology. The identified CVRs consistently featured ventricles in the numerator, suggesting that global changes reflected by ventricular expansion are a crucial component of atrophy monitoring. An interesting advantage of the ventricles is that they are likely summarizing atrophy happening elsewhere, and their size and contrast likely make them a good target for segmentation. The presence of the nucleus accumbens in the denominator across different CVRs might appear more intriguing, but not unexpected: a study by Nie *et al*.^38^ found a correlation between the nucleus accumbens volume and clinical rating scales (MMSE and MoCA).

Although the A4 trial did not meet its primary clinical endpoint, our re-analysis of its imaging data (MRI) provides valuable insights into biomarker sensitivity. Although our analysis did not identify a statistically significant treatment effect for any biomarker (indeed, this difference may not exist), we noted a consistent pattern in which our data-driven CVRs produced considerably larger effect sizes and lower *P*-values than traditional volumetric measures like hippocampus/ICV. This finding is important for two main reasons. First, it suggests that our CVRs may have greater sensitivity to detect subtle, treatment-related signals of slowed atrophy, even in a trial where the overall therapeutic effect was modest or non-existent. This enhanced sensitivity could be critical for future trial design, potentially enabling the detection of a biological effect with smaller sample sizes or shorter durations. Second, it highlights the potential limitations of relying solely on traditional, single-region biomarkers in preclinical or prevention trial settings where expected changes are minimal. The results from this single trial are, of course, exploratory and require confirmation. However, they strongly motivate the usage of these optimized CVRs in pooled analyses across multiple prevention studies or in future trials to definitively establish their added value as sensitive endpoints for measuring disease modification.

Several factors may limit the generalizability and precision of volume-based biomarkers in Alzheimer’s disease trials. First, anti-amyloid immunotherapies have been reported to show accelerated ‘pseudo’ atrophy in the whole brain, hippocampus and ventricles.^44^ This could confound any volumetric biomarker, including CVR, in such a trial. Second, measurement error and segmentation variability in MRI volumetry—stemming from scanner differences, interscan drift or algorithmic bias—are expected to introduce predominantly cross-sectional noise rather than systematic drift and thus would not selectively improve metrics across varied follow-up times. Third, unmeasured lifestyle and clinical confounders (e.g. physical activity, diet, cardiovascular health, concomitant medications) were not uniformly available in our multisite ADNI cohort and may modulate atrophy rates. Fourth, although our sample spans 61 sites and 1381 individuals—factors that mitigate against any single systematic bias—subtle protocol variants or residual comorbidities (e.g. vascular disease, even when the ADNI cohort aimed to exclude major comorbidities) may persist. Nonetheless, since our analysis focuses on biomarker performance comparison in unseen data, any residual confounding is unlikely to significantly bias the comparative findings; irrespective, independent validation or discovery in external cohorts (e.g. A4) will be needed to fully exclude any residual confounding from unmeasured factors. Fifth, the main cohort data have low ethnic diversity; work is underway to rectify this in ADNI^45^ (and elsewhere), but it may be some time before the impact of ethnicity on brain atrophy is fully understood. Importantly, we found in additional robustness analyses (Supplementary Figs 4 and 5) that some regions consistently emerged across random splits, suggesting that any remaining bias would need to act proportionally to scan interval and in the same direction across hundreds of subjects to spuriously enhance all key metrics (group separation, residual variance, SSE). Sixth, no formal quality control was applied to FreeSurfer segmentations. While longitudinal processing reduces variability, residual errors may persist. For such errors to systematically favour CVRs over traditional biomarkers and survive the cross-validation, several unlikely conditions would need to co-occur (see Supplementary Note 2). Our observed metrics suggest CVRs reflect meaningful biological signal rather than amplified noise. Finally, because our framework was applied to data processed by a specific pipeline, investigators should re-run BioDisCVR with their own covariate choices—and, when feasible, on independent datasets—to identify the optimal CVR biomarker for their particular context.

Beyond addressing limitations, three avenues for future work are as follows: first, validating findings on patient-level data from completed clinical trials in Alzheimer’s disease is a clear priority, where smaller effect sizes could be detected on secondary imaging biomarker outcomes. The challenge here is the general reluctance for trial sponsors to share patient-level data, e.g. Clarity-AD (see Supplementary material from van Dyck *et al.*^27^). Indeed, given the notable impact of the clinical trial configuration (Supplementary Table 2), BioDisCVR holds promise for clinical trial design. Second, expanding the CVR framework beyond Alzheimer’s disease into other neurodegenerative disorders or normative modelling contexts could broaden its impact. Third, dissecting the components of ratio-based CVRs by (i) directly comparing CVR performance to the individual regions that comprise each composite and (ii) contrasting both against non-ratio multi-region metrics (e.g. mean or sum of z-scored volumes across relevant regions) to clarify whether gains arise from normalization, regional aggregation or both.

## Conclusions

In conclusion, this study demonstrates the superior performance of CVR biomarkers for monitoring brain atrophy in Alzheimer’s disease. By applying the BioDisCVR framework, originally developed for tau PET, to MRI-based volumetric measures, we have successfully translated the benefits of ratio-based biomarkers to a more widely accessible and cost-effective imaging modality. The enhanced disease signal extraction achieved through CVRs—evidenced by significantly reduced noise and increased group separation—translates directly into tangible advantages for both research and clinical practice, over and above traditional volumetric biomarkers including ventricles, whole brain and hippocampus.

## Supplementary Material

fcaf497_Supplementary_Data
