# Supplementary material for: Enhanced monitoring of Alzheimer's disease brain atrophy using composite value ratios of volumes
Source: Brain Commun. 2025 Dec 15;8(1):fcaf497. doi: 10.1093/braincomms/fcaf497 (PMC12782101; doi:10.1093/braincomms/fcaf497)
Supplement: fcaf497_Supplementary_Data [file fcaf497_supplementary_data.docx]

**Supplementary Figure 1: Flowchart of Participant Inclusion and Exclusion Criteria.** This diagram illustrates the sequential steps taken to derive the final study sample from the initial pool of assessed participants. Of 2361 participants initially assessed for eligibility, 1 participant was excluded due to missing fundamental data. From the remaining 2360, 404 participants were excluded because they had only a single visit, thereby precluding longitudinal analysis. Following this, 575 participants were excluded due to the absence of amyloid-beta (Aβ) data, which was a primary requirement for the study’s biomarker analysis. The final analytical sample comprised 1381 participants, all of whom had complete data for essential covariates, including diagnosis, date of birth (DOB), APOE4 genotype, Mini-Mental State Examination (MMSE) score, sex, and education level.
**
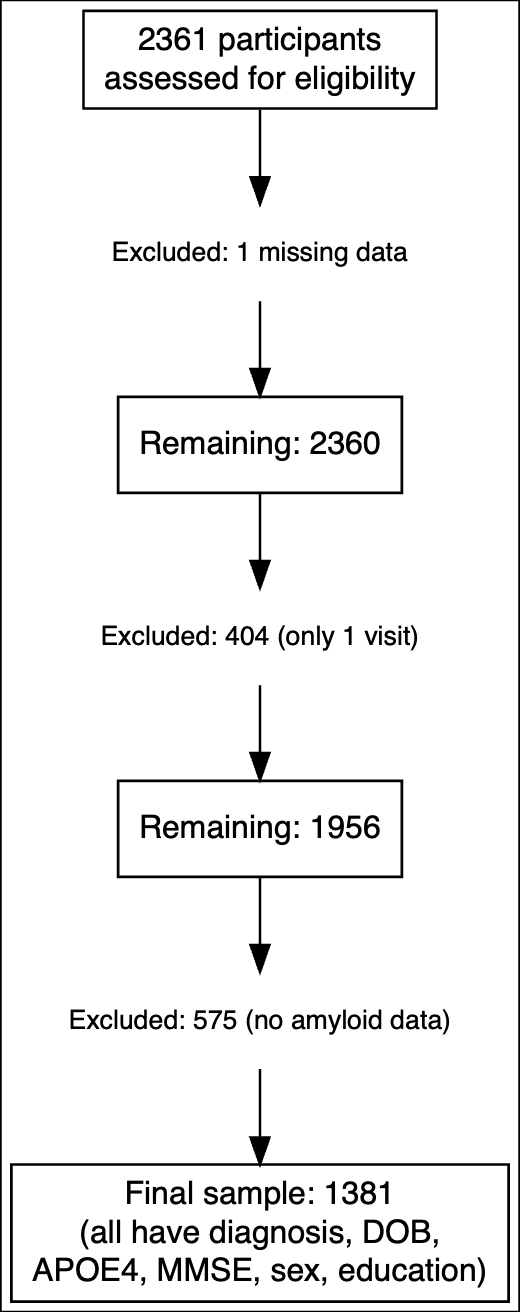
**

**Supplementary Figure 2: Predicted vs. observed values for subset 1 (log‑transformed).** Each point represents a single scan (N = 286 cognitively impaired; N = 49 cognitively unimpaired). CVR denotes the composite value ratio of two data‑driven regions of interest; all other measures are normalised by intracranial volume. CVR naming follows: CI = conditioned to minimise the sample size estimate of cognitively impaired individuals; CU = conditioned to minimise the sample size estimate of cognitively unimpaired individuals; suffix 1 or 2 indicates the discovery subset. The coefficient of determination (R², squared Pearson correlation) is reported in the bottom‑right corner of each subpanel.

**
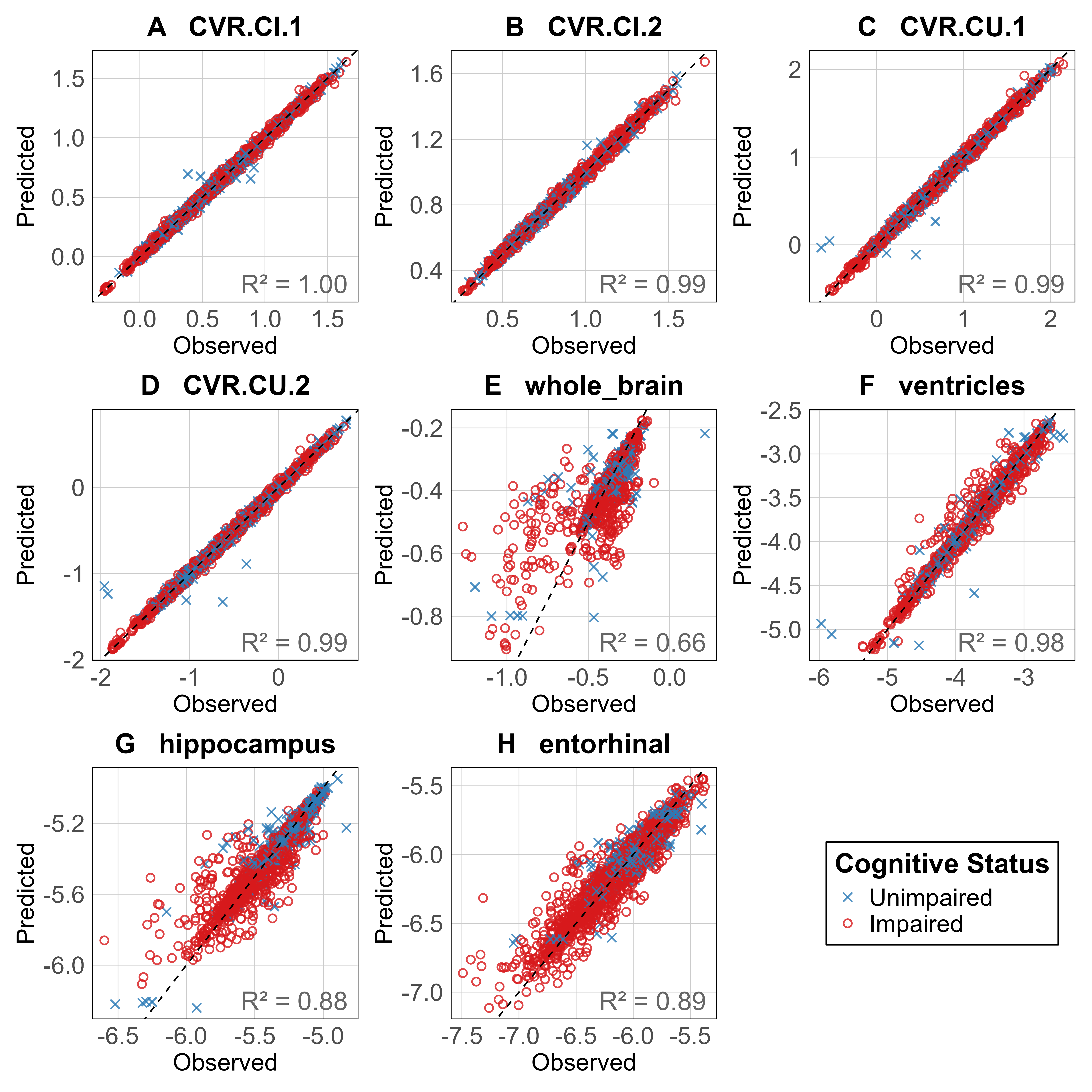
**

**Supplementary Figure 3: Predicted vs. observed values for subset 2 (log‑transformed).** Each point represents a single scan (N = 278 cognitively impaired; N = 39 cognitively unimpaired). CVR denotes the composite value ratio of two data‑driven regions of interest; all other measures are normalised by intracranial volume. CVR naming follows: CI = conditioned to minimise the sample size estimate of cognitively impaired individuals; CU = conditioned to minimise the sample size estimate of cognitively unimpaired individuals; suffix 1 or 2 indicates the discovery subset. The coefficient of determination (R², squared Pearson correlation) is reported in the bottom‑right corner of each subpanel.

**
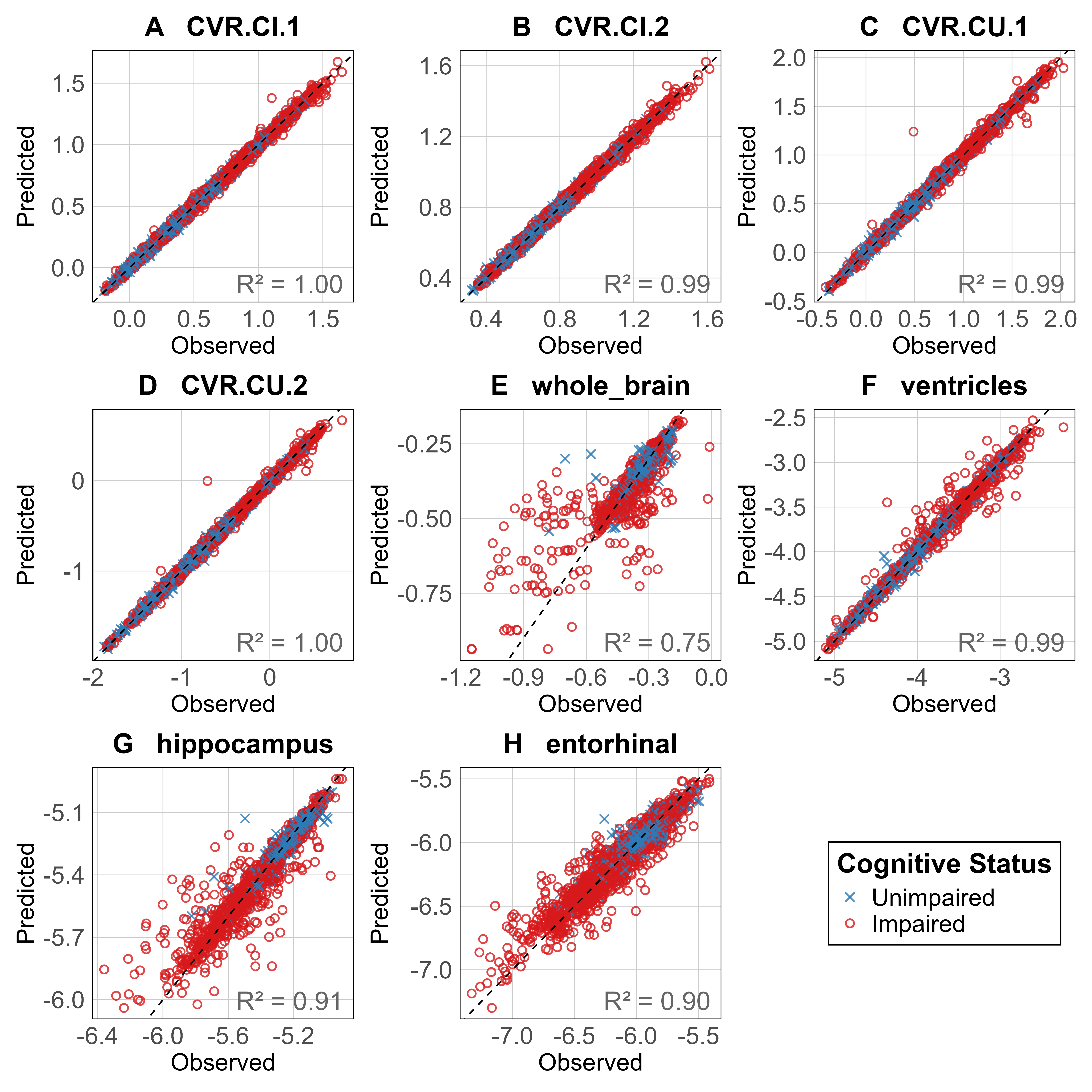
**

**Supplementary Figure 4: Consistent brain region selection within Cognitively Impaired (CI, amyloid-positive) subsamples.** Each stacked horizontal bar represents one brain region, with segments indicating the proportion of runs in which the region was selected as numerator (cyan, left‑to‑right), denominator (magenta, right‑to‑left), or skipped (grey) in constructing the composite value ratio (CVR) biomarker. Results are aggregated across 100 independent BioDisCVR runs, each applied to a random 50% subsample of the CI group (N = 564; ~282 individuals per run). For each region on the y‑axis, the total bar length corresponds to 100% of runs, and the bidirectional x‑axis shows selection percentages for numerator and denominator roles. Recurrent selection patterns highlight the most stable and informative regions for CVR construction within the CI cohort (e.g., ventricles, 100% numerator; hippocampus, 100% denominator).

**
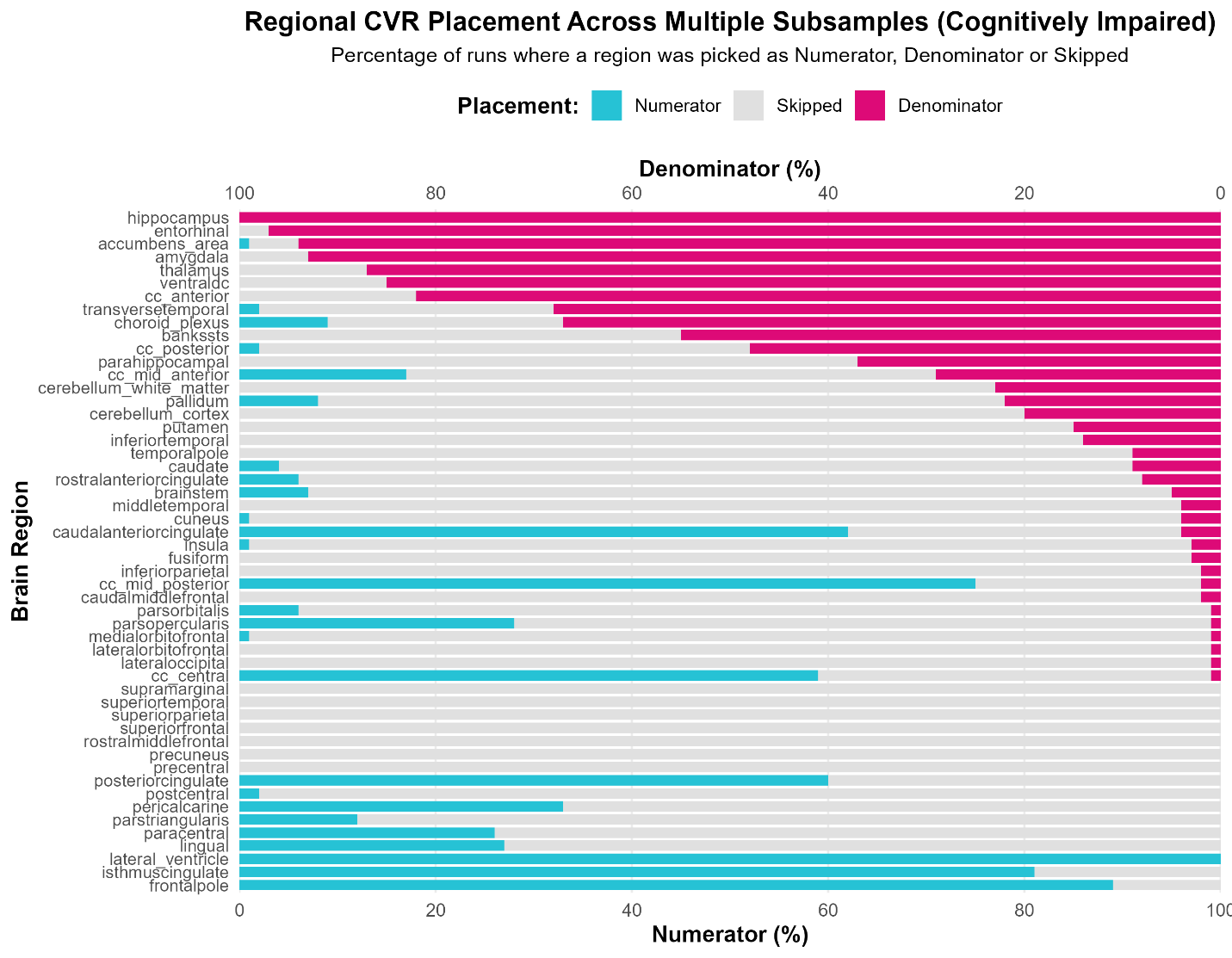
**

**Supplementary Figure 5: Consistent brain region selection within Cognitively Unimpaired (CU, amyloid-positive) subsamples.** Each stacked horizontal bar represents one brain region, with segments indicating the proportion of runs in which the region was selected as numerator (cyan, left‑to‑right), denominator (magenta, right‑to‑left), or skipped (grey) in constructing the composite value ratio (CVR) biomarker. Results are aggregated across 100 independent BioDisCVR runs, each applied to a random 50% subsample of the CU group (N = 88; ~44 individuals per run). For each region on the y‑axis, the total bar length corresponds to 100% of runs, and the bidirectional x‑axis shows selection percentages for numerator and denominator roles. Recurrent selection patterns highlight the most stable and informative regions for CVR construction within the CU cohort (e.g., ventricles, 100% numerator; nucleus accumbens, 82% denominator).

**
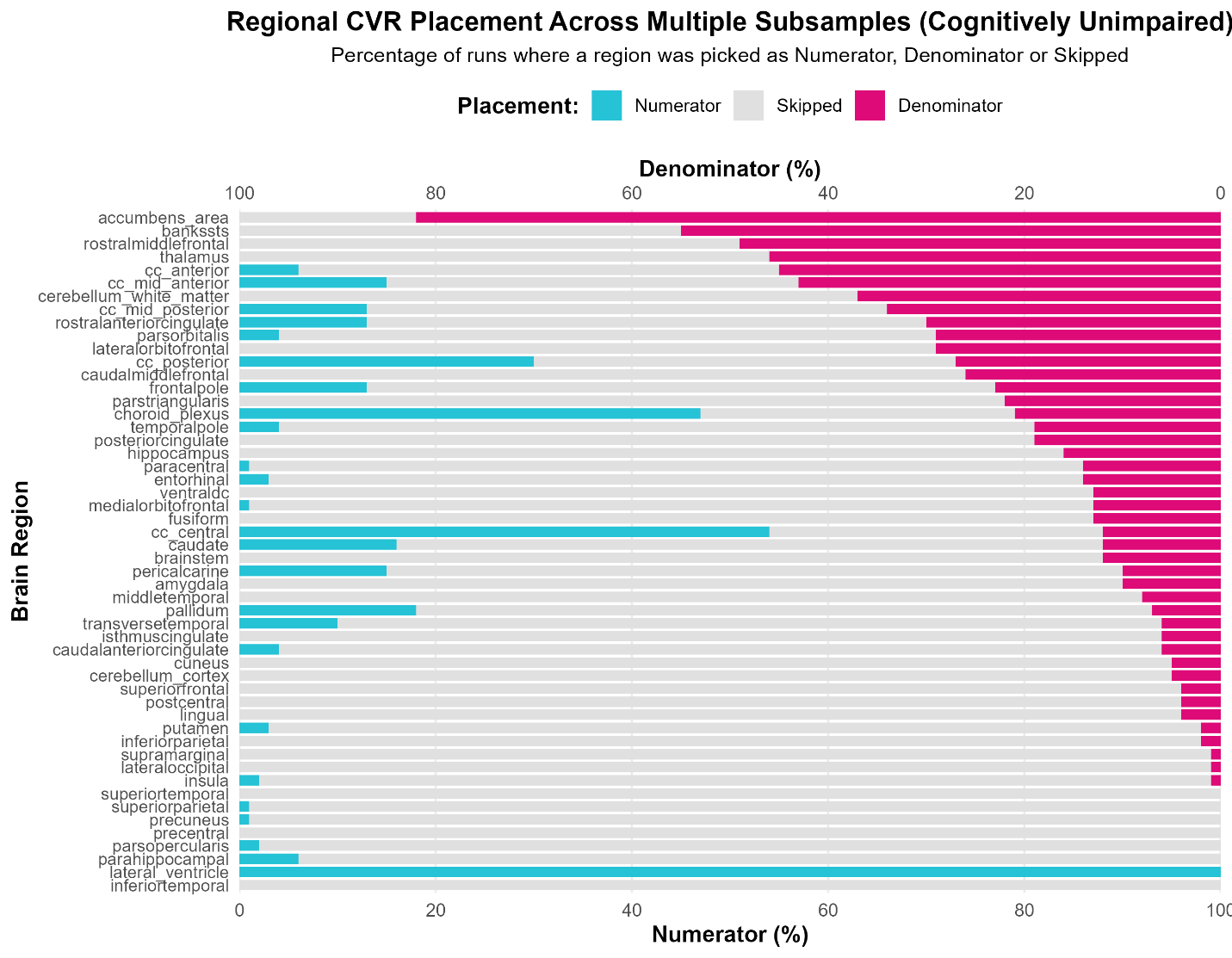
**

**Supplementary Table 1: Race and ethnicity.** Data consisted of different individuals from different backgrounds, albeit predominantly white. Abbreviations: CU = cognitively unimpaired; CI = cognitively impaired; A- = amyloid-negative; A+ = amyloid-positive.

|  | **CU A-** | **CU A+** | **CI A-** | **CI A+** |
| --- | --- | --- | --- | --- |
| Number of individuals | 331 | 88 | 398 | 564 |
| **Race** | | | | |
| American Indian or Alaskan Native | 1 (0.3%) | 0 (0.0%) | 1 (0.3%) | 0 (0.0%) |
| Asian | 5 (1.5%) | 0 (0.0%) | 8 (2.0%) | 7 (1.2%) |
| Black or African American | 24 (7.3%) | 1 (1.1%) | 13 (3.3%) | 18 (3.2%) |
| More than one race | 6 (1.8%) | 4 (4.5%) | 4 (1.0%) | 8 (1.4%) |
| White | 295 (89.1%) | 83 (94.3%) | 368 (92.5%) | 529 (93.8%) |
| Native Hawaiian or Other Pacific Islander | 0 (0.0%) | 0 (0.0%) | 1 (0.3%) | 1 (0.2%) |
| Unknown | 0 (0.0%) | 0 (0.0%) | 3 (0.8%) | 1 (0.2%) |
|  |  |  |  |  |
| **Ethnicity** | | | | |
| Hispanic or Latino | 18 (5.4%) | 4 (4.5%) | 16 (4.0%) | 15 (2.7%) |
| Not Hispanic or Latino | 312 (94.3%) | 82 (93.2%) | 381 (95.7%) | 547 (97.0%) |
| Unknown | 1 (0.3%) | 2 (2.3%) | 1 (0.3%) | 2 (0.4%) |

**Supplementary Table 2: Detectable effect size per example clinical trial.** Given a past clinical trial design (number of participants, trial duration and inclusion criteria), we show the hypothetical detectable effect size per biomarker. The calculation is given by first fitting a linear mixed-effects model to the log-transformed biomarker, with covariates age, sex, APOE4. All classical biomarkers (whole brain, ventricles, hippocampus, entorhinal cortex) are divided by the intracranial volume. Our biomarkers’ names (CVR) indicate the target cognitive group and data subset they have been trained for: CU = cognitively unimpaired; CI = cognitively impaired; suffix .1 and .2 indicate the subset that was used to obtain the biomarker. In bold, for the subset analysis, the best measures per trial, not including the biomarkers that used the same subset data. Asterisk (*) indicates that the region was used for the trial as secondary outcome. Non-CVRs are divided by Intracranial Volume (ICV).

| **All data** | | | | |
| --- | --- | --- | --- | --- |
|  | A4 Study | EMERGE / ENGAGE | Clarity AD | TRAILBLAZER-ALZ 2 |
| CVR.CI.1 | 0.069 | 0.097 | 0.098 | 0.098 |
| CVR.CI.2 | 0.078 | 0.101 | 0.079 | 0.099 |
| CVR.CU.1 | 0.063 | 0.117 | 0.162 | 0.143 |
| CVR.CU.2 | 0.061 | 0.107 | 0.173 | 0.142 |
| *whole brain | 0.354 | 1.495 | 0.659 | 2.444 |
| *ventricles | 0.081 | 0.169 | 0.263 | 0.232 |
| *hippocampus | 0.319 | 0.661 | 0.312 | 1.072 |
| hippo/ventricles | 0.066 | 0.104 | 0.137 | 0.129 |
| entorhinal | 0.289 | 0.467 | 0.268 | 0.486 |
|  |  |  |  |  |
| **Subset 1** | | | | |
|  | A4 Study | EMERGE / ENGAGE | Clarity AD | TRAILBLAZER-ALZ 2 |
| CVR.CI.2 | 0.075 | 0.096 | **0.094** | **0.097** |
| CVR.CU.2 | **0.055** | **0.095** | 0.121 | 0.101 |
| *whole brain | 0.619 | 1.995 | 2.334 | 3.521 |
| *ventricles | 0.089 | 0.170 | 0.180 | 0.206 |
| *hippocampus | 0.654 | 0.721 | 0.609 | 1.250 |
| hippo/ventricles | 0.057 | 0.099 | 0.121 | 0.114 |
| entorhinal | 0.322 | 0.466 | 0.813 | 0.515 |
|  |  |  |  |  |
| **Subset 2** | | | | |
|  | A4 Study | EMERGE / ENGAGE | Clarity AD | TRAILBLAZER-ALZ 2 |
| CVR.CI.1 | **0.077** | **0.112** | **0.108** | **0.114** |
| CVR.CU.1 | 0.079 | 0.133 | 0.169 | 0.183 |
| *whole brain | 0.124 | 1.027 | 0.515 | 1.549 |
| *ventricles | 0.078 | 0.172 | 1.248 | 0.297 |
| *hippocampus | 0.139 | 0.567 | 0.260 | 0.931 |
| hippo/ventricles | 0.078 | 0.114 | 0.142 | 0.154 |
| entorhinal | 0.285 | 0.483 | 0.186 | 0.387 |

**Supplementary Table 3: Association between biomarkers and cognitive measures.** Cross-sectional associations between nine volumetric biomarkers—each standardised to mean = 0 and SD = 1 within the amyloid-positive cohort—and cognitive measures in 1,381 amyloid-positive participants. For each biomarker the table reports the odds ratio (OR) and 95% confidence interval per one standard deviation increase from logistic regression of global CDR status (0 = unimpaired vs > 0 = impaired), the two-tailed p-value, and the area under the receiver operating characteristic curve (AUC) for discrimination, as well as the standardized regression coefficient (β) per one standard deviation increase and corresponding two-tailed p-value from linear regression on baseline MMSE, together with the adjusted R² for variance explained; all models were adjusted for standardized age, sex (0 = male, 1 = female), and standardized years of education.

| **biomarker** | **OR** | **p_logit** | **AUC** | **R2** | **beta** | **p_linear** |
| --- | --- | --- | --- | --- | --- | --- |
| CVR.CI.1 | 1.87 (1.49, 2.36) | 8.54×10^-8^ | 0.703 | 0.147 | -1.06 | 1.91×10^-19^ |
| CVR.CI.2 | 2.1 (1.66, 2.69) | 1.43×10^-9^ | 0.719 | 0.168 | -1.15 | 7.24×10^-23^ |
| CVR.CU.1 | 1.66 (1.34, 2.08) | 5.28×10^-6^ | 0.687 | 0.122 | -0.931 | 2.76×10^-15^ |
| CVR.CU.2 | 1.58 (1.28, 1.97) | 3.16×10^-5^ | 0.681 | 0.106 | -0.853 | 1.02×10^-12^ |
| whole_brain | 1.54 (1.19, 2.07) | 2.39×10^-3^ | 0.671 | 0.0651 | -0.525 | 3.12×10^-6^ |
| ventricles | 1.53 (1.24, 1.89) | 6.77×10^-5^ | 0.679 | 0.107 | -0.846 | 6.56×10^-13^ |
| hippocampus | **2.73 (2.12, 3.57)** | 5.70×10^-14^ | **0.755** | 0.165 | -1.08 | 2.36×10^-22^ |
| ventricles/hippo | 1.96 (1.57, 2.47) | 3.77×10^-9^ | 0.712 | 0.158 | -1.11 | 3.45×10^-21^ |
| entorhinal | 2.08 (1.67, 2.64) | 4.43×10^-10^ | 0.725 | **0.188** | -1.12 | 2.68×10^-26^ |

**Supplementary Table 4: Solanezumab treatment effect on the change of atrophy in the A4 trial cohort.** Least-squares mean difference in the annualised percent change of volumetric biomarkers between the Solanezumab (N=401) and Placebo (N=423) treatment arms. A negative difference indicates less atrophy (treatment benefit) in the solanezumab group compared to placebo. All estimates are derived from Analysis of Covariance (ANCOVA) models adjusted for baseline age, sex (female vs. male), education (≥13 vs. <13 years), ApoE ε4 carrier status, and the baseline value of the biomarker. Values in parentheses represent the 95% confidence interval for the difference. CVR.CU.x = Composite Value Ratio trained on the Cognitively Impaired Subset data (50%) from ADNI; ICV = Intracranial Volume.

| **Biomarker** | **Difference in % change** | **p-value** |
| --- | --- | --- |
| CVR.CU.1 | -0.277 (-0.672, 0.118) | 0.169 |
| CVR.CU.2 | -0.198 (-0.587, 0.191) | 0.318 |
| Ventricles/ICV | -0.132 (-0.504, 0.240) | 0.486 |
| WholeBrain/ICV | 0.0311 (-0.058, 0.120) | 0.494 |
| Hippocampus/Ventricles | -0.0643 (-0.461, 0.332) | 0.751 |
| Hippocampus/ICV | 0.0059 (-0.170, 0.182) | 0.948 |

**Supplementary Note 1: List of available regions.** The following regions were made available for our algorithm: Nucleus accumbens, Amygdala, Banks of the superior temporal sulci, Brainstem, Caudal anterior cingulate gyrus, Caudal middle frontal gyrus, Caudate nucleus, Anterior corpus callosum, Central corpus callosum, Mid-anterior corpus callosum, Mid-posterior corpus callosum, Posterior corpus callosum, Cerebellar cortex, Cerebellar white matter, Choroid plexus, Cuneus, Entorhinal cortex, Frontal poles, Fusiform gyrus, Hippocampus, Inferior parietal lobule, Inferior temporal gyrus, Insular cortex, Isthmus of the cingulate gyri, Lateral ventricles, Lateral occipital cortex, Lateral orbitofrontal cortex, Lingual gyrus, Medial orbitofrontal cortex, Middle temporal gyrus, Paracentral lobule, Parahippocampal gyrus, Pars opercularis, Pars orbitalis, Pars triangularis, Pericalcarine cortex, Postcentral gyrus, Posterior cingulate cortex, Precentral gyrus, Precuneus, Putamen, Pallidum, Rostral anterior cingulate gyrus, Rostral middle frontal gyrus, Superior frontal gyrus, Superior parietal lobule, Superior temporal gyrus, Supramarginal gyrus, Temporal poles, Thalamus, Transverse temporal gyri, Ventral diencephalon.

**Supplementary Note 2: Assessment of potential segmentation biases’ influence.** FreeSurfer is widely used for automated brain segmentation, but known limitations exist. While longitudinal processing reduces variability, segmentation errors may still occur and could theoretically bias volumetric biomarkers.

For segmentation errors to artificially enhance CVR performance over traditional biomarkers, several unlikely conditions would need to simultaneously hold:

1. **Longitudinally Increasing Error**
   Errors would need to grow over time in a way that mimics biological progression. Simple additive or multiplicative biases would cancel out in the CVR structure and be penalised by the linear mixed effects model if they introduced nonlinearity. Our residual metrics do not reflect such penalisation.
2. **Group-Specific Bias**
   Errors would need to disproportionately affect amyloid-positive individuals compared to amyloid-negative ones, thereby artificially enhancing group separation. Random or symmetric bias would dilute rather than amplify the observed effect.
3. **Selective Impact on CVR Regions**
   Errors would have to spare traditional biomarkers (e.g., hippocampus, ventricles, whole brain) while selectively influencing the composite regions used in CVRs. This is implausible given that all regions are derived from the same segmentation pipeline.
4. **Ratio Amplification of Bias**
   The CVR structure would need to magnify these biases in a way that improves longitudinal signal and reduces sample size estimates at a group level. Yet our observed metrics—smaller residuals, stronger group separation, and lower sample size requirements—suggest that CVRs are capturing meaningful biological variance.

Taken together, this constellation of conditions appears highly improbable. Moreover, even if such a systematic bias did exist, if it survives the cross-validation, its consistency and predictive utility would still render it a useful proxy—just as MRI volumes and PET signals serve as indirect but informative biomarkers of underlying pathology. Nonetheless, we acknowledge the importance of formal QC and recommend its inclusion in future studies.
